# Supplementary material for: Irrigation suitability, health risk assessment and source apportionment of heavy metals in surface water used for irrigation near marble industry in Malakand, Pakistan
Source: PLoS One. 2022 Dec 21;17(12):e0279083. doi: 10.1371/journal.pone.0279083 (PMC9770375; doi:10.1371/journal.pone.0279083)
Supplement: S1 Table — (DOCX) [file pone.0279083.s001.docx]

Table S1: Detection limit (LOD), wavelength and % recovery of the analyzed elements

|  |  |  | NIST SRM 1643F and 2781 | | | |
| --- | --- | --- | --- | --- | --- | --- |
| Elements | LOD µg/ml | Wavelength (λ /nm) | Certified Value (µg/L) | Measured Value (µg/L) | % Recovery | % RSD |
| Al | 0.07 | 396.1 | 132.5 ± 1.2 | 132.2 | 99 | 0.7 |
| Ca | 0.01 | 370.6 | 29 140 ± 320 | 29145 | 101 | 1 |
| Cr | 0.02 | 285.4 | 18.32 ± 0.10 | 18.2 | 98 | 0.8 |
| Cu | 0.03 | 324.7 | 21.44 ± 0.70 | 21.1 | 96 | 0.9 |
| Fe | 0.02 | 238.2 | 92.51 ± 0.77 | 92 | 99 | 1.3 |
| K | 0.64 | 766.4 | 1913.3 ± 9.0 | 1915 | 102 | 0.9 |
| Mg | 0.01 | 280.2 | 7 380 ± 58 | 7381 | 101 | 0.5 |
| Mn | 0.01 | 257.6 | 36.77 ± 0.58 | 36 | 98 | 1 |
| Na | 0.05 | 589.5 | 18 640 ± 240 | 18635 | 96 | 1.3 |
| Ni | 0.01 | 231.6 | 59.2 ± 1.4 | 60 | 101 | 1.7 |
| P | 0.05 | 177.4 | 2.43 ± 0.04 | 2.4 | 99 | 1.6 |
| Si | 0.05 | 288.8 | 5.1 ± 0.2 | 4.9 | 99 | 1 |
| Sr | 0.01 | 462.3 | 73.7 ± 1.7 | 74 | 101 | 0.4 |
| Ti | 0.02 | 335.4 | 0.31 ± 0.01 | 0.31 | 100 | 1 |
| Zn | 0.02 | 213.8 | 73.7 ± 1.7 | 73 | 99 | 0.3 |
