# Supplementary material for: Irrigation suitability, health risk assessment and source apportionment of heavy metals in surface water used for irrigation near marble industry in Malakand, Pakistan
Source: PLoS One. 2022 Dec 21;17(12):e0279083. doi: 10.1371/journal.pone.0279083 (PMC9770375; doi:10.1371/journal.pone.0279083)
Supplement: S2 Table — (DOCX) [file pone.0279083.s002.docx]

Table S2: Water quality indices with categories used in the study

| S. No | Water quality assessment parameters | Ranges | | | | | References |  |
| --- | --- | --- | --- | --- | --- | --- | --- | --- |
| 1 | Sodium adsorption ratio (SAR) meq/L | < 10 | 10--18 | 18-26 | > 26 | - | [1] | |
|  | Category | Excellent | Good | Doubtful | Unsuitable | - |  | |
| 2 | Salinity hazard (EC) µs/cm | < 250 | 250-750 | 750-2000 | 2000-3000 | > 3000 | [2] | |
|  | Category | Excellent | Good | Permissible | Doubtful | Unsuitable | [3] | |
| 3 | Sodium percentage (Na%) meq/L | < 20 | 20-40 | 40-60 | 40-80 | > 80 | [4] | |
|  | Category | Excellent | Good | Permissible | Doubtful | Unsuitable |  | |
| 4 | Magnesium adsorption (MAR) meq/L | < 50 | > 50 | - | - | - | [5] | |
|  | Category | Suitable | Unsuitable | - | - | - |  | |
| 5 | Kelly ratio (KR) meq/L | < 1 | > 1 | - | - | - | [6] | |
|  | Category | Fit | Unfit | - | - | - |  | |

References

1. Richards L. . Diagnosis and Improvement of Saline and Alkali Soils. Soil Sci. 1954;78. Available: https://journals.lww.com/soilsci/Fulltext/1954/08000/Diagnosis_and_Improvement_of_Saline_and_Alkali.12.aspx

2. Raghunath HM. Geochemical survey and water quality. New Delh: Groundwater Wiley eastern limited; 1987.

3. Alfaifi H, El-Sorogy AS, Qaysi S, Kahal A, Almadani S, Alshehri F, et al. Evaluation of heavy metal contamination and groundwater quality along the Red Sea coast, southern Saudi Arabia. Mar Pollut Bull. 2021;163: 111975. doi:10.1016/j.marpolbul.2021.111975

4. Xiao J, Wang L, Deng L, Jin Z. Characteristics, sources, water quality and health risk assessment of trace elements in river water and well water in the Chinese Loess Plateau. Sci Total Environ. 2019;650: 2004–2012. doi:10.1016/j.scitotenv.2018.09.322

5. Jehan S, Ullah I, Khan S, Muhammad S, Khattak SA, Khan T. Evaluation of the Swat River, Northern Pakistan, water quality using multivariate statistical techniques and water quality index (WQI) model. Environ Sci Pollut Res. 2020;27: 38545–38558. doi:10.1007/s11356-020-09688-y

6. Kelley WP. Use of saline irrigation water. Soil Sci. 1963;95: 385–391.
