# Supplementary material for: Irrigation suitability, health risk assessment and source apportionment of heavy metals in surface water used for irrigation near marble industry in Malakand, Pakistan
Source: PLoS One. 2022 Dec 21;17(12):e0279083. doi: 10.1371/journal.pone.0279083 (PMC9770375; doi:10.1371/journal.pone.0279083)
Supplement: S3 Table — (DOCX) [file pone.0279083.s003.docx]

**Table S3:** Parameters used for calculation of noncarcinogenic and carcinogenic dermal risk assessment

|  |  | Group | |  |  |  |
| --- | --- | --- | --- | --- | --- | --- |
| Parameter | Description | Adult | Child | Unit | Reference | |
| Cw | The average concentration of heavy metals in a water sample | - | - | mg/L | This study | |
| SA | The surface area of the skin | 18000 | 6600 | cm^2^ | [1] | |
| EF | Exposure frequency | 350 | 350 | days/year | [1] | |
| ED | ED is exposure duration which for this study | 70 | 6 | years | [2] | |
| BW | Average body weight | 70 | 15 | Kg | [1] | |
| AT | Average exposure time | 365 × ED | - | days | [1] | |
| ET | Exposure time during bathing and shower/irrigation | 0.58 | 1 | h/day | [1] | |
| CF | Unit conversion factor | 0.001 | 0.001 | L/cm^3^ | [1] | |
| Kp | Dermal permeability coefficient | Al, Cu, Fe, Mn = 0.001,  Cr, 0.002, Ni= 0.004,  Zn = 0.0006 | | cm/h | [1] | |
|  |  |  |  |  |  | |
| SF | Slope factor | Cr = 0.5, Ni = 0.9, |  |  | [3] |  |
| IR | Ingestion rate | 2.0 | 0.64 | L/day | [4] |  |

References

1. USEPA. Risk Assessment Guidance for Superfund Volume I : Human Health Evaluation Manual ( Part E , Supplemental Guidance for Dermal Risk Assessment ) Final. 2004.

2. Saleem M, Iqbal J, Shah MH. Seasonal variations, risk assessment and multivariate analysis of trace metals in the freshwater reservoirs of Pakistan. Chemosphere. 2019;216: 715–724. doi:https://doi.org/10.1016/j.chemosphere.2018.10.173

3. Song T, Chen Y, Du S, Yang F. Hydrogeochemical evolution and risk assessment of human health in a riverbank filtration site, northeastern China. Hum Ecol Risk Assess. 2017;23: 705–726. doi:10.1080/10807039.2016.1277413

4. Njuguna SM, Onyango JA, Githaiga KB, Gituru RW, Yan X. Application of multivariate statistical analysis and water quality index in health risk assessment by domestic use of river water. Case study of Tana River in Kenya. Process Saf Environ Prot. 2020;133: 149–158. doi:10.1016/j.psep.2019.11.006
