# Supplementary material for: Irrigation suitability, health risk assessment and source apportionment of heavy metals in surface water used for irrigation near marble industry in Malakand, Pakistan
Source: PLoS One. 2022 Dec 21;17(12):e0279083. doi: 10.1371/journal.pone.0279083 (PMC9770375; doi:10.1371/journal.pone.0279083)
Supplement: Attachment — Submitted filename: Response to Reviewers.docx [file pone.0279083.s004.docx]

**PONE-D-22-06684 - [EMID:a449285f229d39f0]**

**Response to Reviewers**

**Q.1: Is the manuscript technically sound and do the data support to the conclusion?**

**Answer:**

The study area is divided into three experimental units in the following way: (i) Control (C) (ii) Industrial sites (S1-S7) and downstream (S8). The data was collected in Replication with quite good sample size. The appropriated conclusion from the data sat was drawn and written in the manuscript

**Q.2: Has the statistical analysis been performed appropriately and rigorously?**

**Answer:** Yes. Descriptive and compositional data analysis tools were used for good interpretation of the data set.
