# Supplementary material for: Irrigation suitability, health risk assessment and source apportionment of heavy metals in surface water used for irrigation near marble industry in Malakand, Pakistan
Source: PLoS One. 2022 Dec 21;17(12):e0279083. doi: 10.1371/journal.pone.0279083 (PMC9770375; doi:10.1371/journal.pone.0279083)
Supplement: Attachment — Submitted filename: Response to Reviewers.docx [file pone.0279083.s005.docx]

To,

Editor-in-Chief

PLOS ONE

September 15, 2022

Dear sir,

I am pleased to resubmit the revised manuscript entitled **“Irrigation suitability, health risk assessment and source apportionment of heavy metals in surface water used for irrigation near marble industry in Malakand, Pakistan’’ ID PONE-D-22-06684R1**.

Sir, I am thankful to the reviewers and academic editors for their valuable time and useful contribution, and highly appreciate the inputs they have given and that their inputs helped in the improvement of the manuscript. The response to the reviewer's comments is contained in the next few paragraphs of this letter. The author is requested for full fee waiver as the manuscript is part of my Ph.D. study and was not funded by any organisation. Moreover, our country is severely hit by the recent flood. Your cooperation will be highly appreciated

**Asghar Khan**

Department of Botany,

Islamia College Peshawar, Pakistan

**Email:** malakand35@gmail.com

**Response to reviewer comments**

The reviewers’ comments on points 2 to 5 unanimously recommend the manuscript for acceptance. In addition, responses to the proposed changes by the reviewers from point six (6) onwards are discussed in detail in the proceeding paragraphs.

**2. Is the manuscript technically sound, and do the data support the conclusions?**

Reviewer #2: Yes

Reviewer #3: Yes

**3. Has the statistical analysis been performed appropriately and rigorously?**

Reviewer #2: Yes

Reviewer #3: Yes

4. Have the authors made all data underlying the findings in their manuscript fully available?

Reviewer #2: Yes

Reviewer #3: Yes

5. Is the manuscript presented in an intelligible fashion and written in standard English?

Reviewer #2: Yes

Reviewer #3: Yes

Reviewer #2:

6.  Alternative irrigation water sources are needed due to the decrease in available clean water resources. One of the best alternatives among these is the use of marginal waters such as wastewater. For this purpose, studies should be supported. In addition, field studies are important in terms of both time and effort. In the study, sampling, analysis and writing stages are sufficient.

**Answer:**

Using wastewater for irrigation is the best alternative source of irrigation. We discussed this alternative source of irrigation with a slight modification in the manuscript in lines (60-65:76-78:103-110:116-123). Reusing wastewater for irrigation alleviates the intense pressure on freshwater extraction and provides nutrients to plants and crops but without proper management (treatment), wastewater can affect plant growth, human health, general economic conditions of the community, and even the cultural and religious values of human society as wastewater contains pathogens (bacteria, viruses, protozoa, and molds) and potentially toxic elements in excessive concentrations. Moreover, the reviewer endorsed that the current study sampling, analysis, and writing stages are sufficient.

**Reviewer #3:** 
General comments
1. In this study, authors evaluated heavy metals (HM) levels in surface water nearby a marble industry for irrigation suitability purposes together with associated health risks in Malakand, Pakistan. Using GIS, authors collected 27 samples of surface water used for irrigation of crops and analyzed for heavy metals (Al, Ca, Cr, Cu, Fe, K, Mg, Mn, Na, Ni, P, Si, Sr, Ti, and Zn) through inductively coupled plasma optical emission. Using heavy metal contents, authors calculated health risks through ingestion and dermal pathway. Results of the Kelly’s ratio (KR) classified the surface water as unsuitable for irrigation. The average daily absorbed dose (ADD), total hazard quotient (THQ > 1) and hazard index (HI > 1) revealed considerable risk of noncarcinogenic toxicity for aduslts and children for certain heavy metals through the ingestion and dermal exposure routes. Especially, cancer risk factor values for Cr and Ni were also higher than the USEPA threshold revealing potential carcinogenic risk. Authors also performed multivariate analysis [CoDa PCA, PCA biplot (clr)]. Authors proposed regular monitoring and remediation strategies of studied waters before being used for irrigation.

**Answer:** The reviewers' comments are clear and support the acceptance of the manuscript for publication.

2. Overall, manuscript contains useful data and can be a good contribution to scientific literature. Study plan is sound accompanied with the use of latest statistical analyses techniques such as PCA, which have made this study impressive. However, I recommend major revisions of manuscript based on following comments.

**Answer:** The reviewer's comments are clear and recommend the manuscript for publication with major revisions. The revision was made following the reviewers' comments and the responses were included in the next sections.

3. Manuscript needs language improvement. There are several sentences which contain either grammatical error or are not easy to follow/understand.

**Answer:** The manuscript has been completely revised; the grammatical errors have been rectified. The long sentences have also been revised.

4. Abstract needs revision. Currently, abstract is very general without any quantitative data. Methodological detail is missing in abstract. Authors may include number of samples collected in abstract. Authors may also present here the % of samples having heavy metal levels above limit values.

**Answer:** The abstract has been revised. Quantitative data in terms of samples and percent results are included. Methodological details were also revised and well explained**.**

5. Introduction sections well-explains the study background and objectives. However, it needs language improvement. Moreover, authors may include some more data here about industrial water treatment and wastewater irrigation concept of the country. For reference, author may consult following review article about wastewater production, composition and use for irrigation in the country.

**Answer:** Language of the introduction section was improved. The data on industrial water treatment was included (lines: 111-115, 525-529) and wastewater irrigation concept of the country was also well explained (lines: 60-66, 103-109,116-123).

6. A critical analysis of wastewater use in agriculture and associated health risks in Pakistan. Environmental Geochemistry and Health. [doi.org/10.1007/s10653-020-00702-3](http://doi.org/10.1007/s10653-020-00702-3)

7. A review of environmental contamination and health risk assessment of wastewater use for crop irrigation with a focus on low and high income countries. International Journal of Environmental Research and Public Health. 15(5), 895

**Answer:** About wastewater production, composition and use for irrigation in the country. The author fully read the above two articles and extracted the concept and presented well in the introduction section of the manuscript (lines 81-93;105-110)

8. Reference citation is vague and needs careful attention. Authors must follow journal style and format for citations. Currently, authors have cited article by numbers and name of authors. For example:
• Line 155: The people of the study area are primarily connected with agricultural sector that grows various cereal crops and vegetables (Wahid et al., 2017).
• Line 229: (Aitchison, 1982; Egozcue et al., 2003; Egozcue and Pawlowsky-Glahn, 2005).
• Line 153: According to (44)

**Answer:** The reference citations have been corrected according to the Journal style and format. The citation errors highlighted the lines number (line155; Line 229 and Line 153) have been reformatted.

9. Moreover, authors have cited a total of 142 references in this which are too many for a research article. Please cite only necessary reverences and limit the reference count to < 40.

**Answer:** The author completely agrees with the reviewer's suggestions to reduction of references. The references were reduced to 129. All the cited references are very important and relevant that logically support the manuscript. Further reduction in references may lead to biased opinions.

10. Methodology is well-described. Study area is well-explained, and all the risk indices are well-presented with proper references. However, Statistical analysis is too long and contains unnecessary detail and too many references.

**Answer:** The statistical analysis was revised and the unnecessary details with too many references were deleted.

11. What is the justification/reason of selecting the following heavy metals (metals (Al, Ca, Cr, Cu, Fe, K, Mg, Mn, Na, Ni, P, Si, Sr, Ti, and Zn) for analysis in this study? Why of some other important heavy metal(loid)s such as As, Cd, Pb etc.

Answer: The samples were analyzed by ICP-OES with a special focus on heavy metal(loid)s such as As, Cd, Pb as well. The metal(loid)s such as As, Cd, Pb was not detected by the machine and will include in the future study.

12. Results and discussion section seems ok. It would be better to concise this section if possible. The introductory sentences in each section, which are basically repetitions, may be deleted. For example, Line 461-462: “Assessment of human health risk is an approach widely used to compute the nature and probability of hostile health effects in humans (124).”.

Answer: The result and discussion section were concise and the highlighted repetitions in Lines 124 and 461-462 were deleted

13. Conclusion seems like abstract. Here, authors must present their concluding results and recommendations rather than just repeating abstract.
**Answer**: The conclusion was revised, and the concluding results and recommendations were well presented (Line 515-533).

14. Authors have used several abbreviations in the manuscript. However, the use of abbreviations is not as per rule. Generally, an abbreviation must be used at the first appearance of full name in the text and then the abbreviation must be used throughout the manuscript. Here authors have used both randomly.

**Answer:** Abbreviations in the manuscript have been rectified according to the Journal guidelines

15. Following are some examples of sentences needing grammatical revision or are not easy to understand/follow. Entire manuscript needs grammatical revision.
• Line 22-23: Health risks from HM exposure were calculated through ingestion and dermal pathway by application …..
• Line 32-34: Estimated Incremental lifetime cancer risk values for Cr and Ni were also higher than the USEPA threshold of 1 x 10−6 - 1 x 10−4 revealed potential carcinogenic risk.
• Line : The current study results revealed both non-carcinogenic and carcinogenic health risks to the exposure population.
• Line 37-38: Therefore, regular monitoring and remediation strategies is ….
• Line 55: Industrialization and urbanization process is beneficial….
• Line 55-58: Too long sentence to follow. Please use small and easily understandable sentences: “Industrialization and urbanization process is beneficial, particularly in the aspect of shaping demographic characteristics, however the rapid, unplanned, random and uncontrolled conversion of the tangible landscape may result undesirable change on water and land ecosystem in terms of deterioration and scarcity of fresh water, posing an extensive threat to global food security (1–3).”
• Line 83-84: Globally the issue of heavy metal (HM) pollution of fresh water resources getting more attention due to the toxic, persistence and bioaccumulative nature (19)
• Line 153: According to (44) the supplementary contributor of pollutant load to the irrigation channels in study area were surface runoff, urban wastewater, and atmospheric deposition of heavy metals.

**Answer:** The large sentences lines no**. (**22-23; 32-34; 37-38; 55; 55-58;83-84; and 153) have been completely revised, grammatically corrected and understandable.

I hope responses to the reviewers’ comments will be helpful in further processing of the manuscript. In case of any discrepancies the authors are happy to respond them and will be much thankful to the editorial team.

**Asghar Khan**

Department of Botany,

Islamia College Peshawar, Pakistan

**Email:** malakand35@gmail.com
